# “Life after treatment”: what physicians consider important for oral cancer survivors’ quality of life

**DOI:** 10.3389/fpubh.2026.1805929

**Published:** 2026-04-13

**Authors:** Vikram Niranjan, Sudarshan Ranpise

**Affiliations:** 1 School of Medicine, Health Research Institute, University of Limerick, Limerick, Ireland; 2 BVP Dental College, Navi Mumbai, Maharashtra, India

**Keywords:** head and neck oncology, India, oral cancer, physician perspectives, postoperative care, qualitative research, quality of life, rehabilitation

## Abstract

**Introduction:**

Oral cancer remains a major public health concern in India, particularly in Maharashtra, where tobacco and areca-nut use, delayed diagnosis, and socioeconomic constraints contribute to high morbidity and poor survivorship outcomes. Post-treatment challenges—including impairments in mastication, swallowing, speech, appearance, emotional wellbeing, and financial stability—profoundly shape survivors’ quality of life (QOL). Physicians’ perspectives are central to survivorship planning, yet remain understudied in low-resource, high-burden settings.

**Methods:**

This qualitative study explored treating physicians’ perceptions of postoperative QOL among oral cancer survivors in Aurangabad, Maharashtra. Semi-structured interviews were conducted with twelve physicians across surgical, medical, dental, and psychosocial specialties. Interviews were audio-recorded, transcribed verbatim, and analyzed using the Framework Method with combined inductive–deductive coding.

**Results:**

Five major themes were identified: (1) physicians’ conceptualizations of QOL; (2) key affected domains, including physical, emotional, social, and financial dimensions; (3) perceived patient needs, such as health education, financial assistance, and family support; (4) barriers to optimal QOL, including clinical sequelae, psychological insecurity, prosthetic challenges, and limited follow-up; and (5) strategies to enhance QOL, including structured health education, coping and psychological support, dietetic guidance, reconstructive rehabilitation, and regular follow-ups. Physicians viewed QOL as a multidimensional construct influenced by functional recovery, psychosocial resilience, and socioeconomic context.

**Discussion:**

Findings highlight the need for integrated survivorship pathways that prioritize multidisciplinary rehabilitation, psychological care, financial navigation, and patient-centered education. Strengthening survivorship infrastructure in resource-constrained settings may substantially improve long-term QOL for oral cancer survivors in India.

## Introduction

Oral cancer constitutes a major global public health burden, with India bearing one of the highest incidence and mortality rates worldwide, with approximately 130,000 new cases and more than 75,000 deaths in 2020 alone, ranking it as the top cancer among males (1, 2). A substantial proportion of global cases is attributable to smokeless tobacco and areca-nut consumption, behaviors particularly prevalent in South-Central Asia. Recent global estimates indicate that India contributes the largest national share of oral cancer cases, reflecting persistent exposure to high-risk habits, delayed diagnosis, and structural inequities in access to care (3). In addition to tobacco and areca-nut use, key determinants of oral cancer in India include alcohol use, human papillomavirus (HPV) infection, older age, male sex, and socioeconomic deprivation (4, 5).

Although advances in surgery, radiotherapy, chemotherapy, and reconstructive techniques have improved survival, many survivors continue to experience long-term impairments in physical, psychosocial, and functional domains. Empirical studies consistently show that postoperative survivors experience persistent difficulties with mastication, swallowing, mouth opening, xerostomia, speech, facial disfigurement, and social withdrawal, which together contribute to diminished health-related quality of life (HRQoL) (6–8). Longitudinal research from India likewise demonstrates that emotional functioning, pain, and swallowing often worsen after treatment, with only partial improvement over time (9, 10). Even after dental or implant-supported prosthetic rehabilitation, survivors may continue to experience functional limitations; however, improved appearance, self-esteem, and social confidence are frequently reported (11).

Most existing research focuses on patient-reported QOL outcomes (12). Far less is known about how physicians themselves conceptualize and assess QOL, despite clinicians’ perceptions directly shaping treatment planning, postoperative counseling, referrals for rehabilitation, and patterns of clinical follow-up (13). Evidence from other cancers suggests that clinicians’ perceptions do not always align with patient-reported outcomes. For example, findings from the SOAPP study showed that clinicians rated lung cancer patients as having poorer QOL than breast, colon, or prostate cancer patients—even after adjustment for disease stage and performance status—highlighting potential clinician perception bias (14). This underscores the value of investigating how physicians interpret QOL in cancers with high functional morbidity, such as oral cancer.

A significant knowledge gap persists regarding clinicians’ perspectives on postoperative QOL among oral cancer survivors in high-burden, low-resource contexts such as India (15, 16). Understanding physicians’ views is especially important in regions like Aurangabad, Maharashtra, where tobacco and areca-nut habits, delayed care-seeking, and socioeconomic challenges amplify survivorship vulnerabilities. Physicians in such settings often make decisions about reconstruction, prosthetic referral, nutritional counseling, psychosocial support, and dependence on caregivers—decisions inherently influenced by their own assumptions about QOL (17).

This study addresses this gap by qualitatively exploring treating physicians’ perceptions of postoperative QOL among oral cancer survivors in Aurangabad. By examining how clinicians conceptualize QOL, identify affected domains, assess rehabilitation needs, recognize barriers, and recommend strategies for improvement, the study provides context-specific insights that can inform the design of integrated survivorship pathways tailored to Indian healthcare settings.

## Methods

### Aim

The study aimed to explore treating physicians’ perceptions of postoperative QOL among oral cancer survivors.

### Objective

To examine how physicians evaluate patient prognosis relative to perceived QOL during the postoperative survivorship period.

### Study design and setting

A qualitative descriptive design was adopted to elicit physicians’ interpretations, clinical judgments, and experiential insights regarding postoperative QOL. Semi-structured, in-depth interviews were conducted with physicians across multiple public and private hospitals in Aurangabad, Maharashtra.

### Participant recruitment

A purposive sampling strategy was used to recruit physicians involved in the diagnosis, surgical treatment, postoperative care, or rehabilitation of patients with oral cancer. Eligible participants included oral and maxillofacial surgeons, oncologists, plastic surgeons, prosthodontists, psychiatrists, and general physicians. The sample size of 12 clinicians aligns with recommendations for qualitative research, achieving data saturation in exploratory studies, where 6–12 interviews typically suffice for identifying core themes across multidisciplinary perspectives, as confirmed by iterative analysis showing theme stabilization by the final interviews.

### Data collection

A semi-structured interview guide informed by QOL frameworks and clinical literature (18, 34) was used. The guide explored postoperative functioning, psychosocial wellbeing, rehabilitation needs, barriers to recovery, and physicians’ conceptualizations of QOL.

The interviews were conducted at each clinician’s clinic/hospital via a prior appointment during non-working hours to avoid any disturbances impacting the process. Each interview lasted approximately 30 min and was conducted in a private hospital setting. With participants’ consent, all interviews were audio-recorded. Each file was assigned a unique identifier and stored securely with encrypted backup. Recordings were transcribed verbatim in English. Transcription accuracy was ensured through iterative verification against audio files and checks for formatting consistency. Field notes documenting contextual cues and observations were appended to transcripts.

### Data management and coding

Transcripts were imported into ATLAS.ti Version 7 (ATLAS.ti Scientific Software Development GmbH, Germany) for management and coding. A combined deductive–inductive approach was used following the Framework Method (18, 34).

A preliminary codebook was developed *a priori* based on study objectives and conceptual domains. This codebook was iteratively refined through team discussions. Intercoder reliability was enhanced through periodic joint-coding sessions. Disagreements were resolved through consensus, and revisions were incorporated into the evolving codebook. All coded transcripts, memos, and analytic notes were organized within a dedicated Hermeneutic Unit (HU) for systematic retrieval and analysis. This qualitative study reached thematic saturation at the level of the overall dataset, with no new codes or themes emerging in the final interviews despite the inclusion of multiple specialties. Given the relatively small number of participants in each specialty, saturation was not assessed separately by discipline; therefore, the findings should be interpreted as reflecting shared cross-disciplinary perspectives rather than specialty-specific views. The coding process involved two researchers who independently open-coded the first 50% of transcripts (six interviews), achieving 87% agreement on initial codes, followed by joint coding of the remaining transcripts to iteratively refine the codebook. Disagreements were resolved through structured discussion sessions documented in an audit trail in which the lead researcher facilitated consensus by referring back to raw data excerpts and established coding criteria, ensuring interpretive consistency without relying on statistical measures such as Cohen’s kappa, which are less suitable for qualitative thematic analysis.

### Analytical framework and thematic development

Framework analysis guided data interpretation. Five thematic areas were identified through iterative refinement: Understanding of QOL, affected domains of QOL, Patients’ needs, Barriers to optimal QOL, and Strategies to overcome barriers.

The analytical framework included themes, categories, and subcategories derived from deductive expectations and inductive insights. Meaningful excerpts were charted across thematic matrices to support cross-case comparisons and pattern recognition. Themes were synthesized into interpretive summaries supported by illustrative quotations, forming the basis of the Results section. The research team employed researcher reflexivity by documenting positionality statements and engaging in regular reflective discussions to mitigate potential biases arising from clinical backgrounds. Participant selection utilized purposive sampling with criterion and maximum variation strategies to ensure representation across specialties, while trustworthiness was established through prolonged engagement, thick descriptive quotes, audit trails of coding decisions, and triangulation across participant responses and emerging themes.

### Ethical considerations

Ethical approval was granted by the Research & Ethics Committee of NCRD’s Sterling Institute of Pharmacy, Navi Mumbai. Written informed consent was obtained from all physicians before data collection. Participant confidentiality was ensured through de-identification and secure data handling.

## Results

The study included 12 physicians (7 male, 5 female) from diverse specialties, all practicing exclusively in private hospitals across urban India, with professional experience ranging from 5 to 20 years (median: 12 years). This gender distribution reflects reasonable representation without notable bias. However, the exclusive recruitment from private settings may limit insights from public-sector contexts where resource constraints and patient demographics differ significantly. Participants comprised oral and maxillofacial surgeons (*n* = 2), oncologists (*n* = 2), plastic surgeons (*n* = 2), prosthodontists (*n* = 2), and one psychiatrist, one oncologist, one general surgeon, and one general medicine specialist, ensuring multidisciplinary perspectives on oral cancer survivorship care. Analysis yielded five major themes, each comprising categories and subcategories that reflect physicians’ perspectives on postoperative QOL among oral cancer survivors.

### Theme 1: understanding of quality of life

This first theme attempts to capture the concept and definition of QOL among the doctors who have treated the oral cancer patients. Mostly, doctors’ opinions about QOL were based on their clinical practice experiences. When viewed in the context of post-operative QOL in oral cancer patients, a notable opinion was that QOL is a process involving multiple aspects working together across different components of a health care system (tolerance to surgery, radiotherapy, human resources, financing, emotions, etc.).

#### Concept

The term ‘QOL’ was subject to multiple interpretations by doctors and was often used both ambiguously and interchangeably with words like ‘disease-free,’ ‘perfect health,’ ‘clinical health,’ and ‘functional health.’ The general awareness of the concept was based on their own understanding rather than theoretical knowledge. However, their understanding was consistent with the concept of QOL given in theory. When asked about their knowledge of QOL, we have the following replies:

“In post-treatment QOL, almost 50% of the patients are psychologically crippled. Probably, though they have undergone surgery, chemo, radiation, and all, functionally, they are not 100%. Only 70% of patients are functional. Maybe less than that. So, it is not good as we compare it with the normal people.”—Doctor 1

“I think what I mean or what I understand by the QOL of patients who have been operated for cancer is related to their comfort or physical comfort related to their day-to-day functions. In the context of oral cancer, I think the important thing is that they can open their mouth properly, which means there should be no trismus. Second, they should be able to masticate or chew the food properly, whether they are taking medication after treatment or after surgery. Third, they should swallow properly, or deglutition. There should not be dysphagia, either with pure surgery or surgery with radiotherapy. Fourth, I would like to discuss the cosmetic appearance of the face, head, and neck area. They should not have scars that look ugly, bad, which probably should not limit their social participation, I am not saying social acceptance, but patients themselves should feel that they are normal individuals. That is how I look at the QOL. Fifth, I would like to point out that they should try to resume whatever duties they were performing before. This is how I look at the total QOL.” —Doctor 2

“Oral Cancer patients mainly presented in late stages to us, after referral by an oral surgeon, for counseling purposes or if they are suffering from depression or any sort of psychological issues. Their QOL is very poor because they have lots of issues related to cancer, cancer treatment, and cancer-related complications. So, their psychological issues are that they are under tremendous stress, such as anxiety, and the fear of death is more common among cancer patients. Depression and insomnia are very common in these patients. In some patients, they present with psychosis when they come to know that they are suffering from cancer, which is a very shocking thing for them.” —Doctor 3

#### Definition

Several doctors included other health determinants in defining their knowledge and understanding of QOL, integrating clinical, social, functional, and emotional factors. ‘QOL’ was also understood as a unifying factor that could potentially align individual health with different clinical parameters, wherein various stages of a postoperative cancer life spanned. Doctors have stated that they have read a few articles on oral cancer and QOL, which brought to light the understanding and knowledge.

“Few articles are there which belong to…now see, the timing and change in the surgical module, probably now we have better surgical rehabilitation options available in last 5 years or may be 10 years, last decade, but if we see our patients that have been operated by us only when or maybe they operated with local flaps and only resection part the quality is not good. Even literature suggests that these patients are socially crippled patients and they stick to a room, they will not have social life, they are alone, they are depressed… they need psychological help, they have psychological problems, dependent on other people, so literature does say it.” —Doctor 4

A few doctors said their perception of QOL is based on their clinical practice experience.

“I really have not gone through the article, but this is my personal perception, that is how I look at the points related to patients in my clinical practice.” —Doctor 5

“It is based on my patients’ experience and also when we read about oncology & psychological issues related to it, so both the things.” —Doctor 6

### Theme 2: affected domains of quality of life

There was a consensus that the different domains of QOL are equally important which are intergral to improve health. We asked the doctors about their knowledge and experience regarding oral cancer patients, with a focus on post-operative management. Doctors enlisted symptoms such as pain, nausea, vomiting, hair loss, mouth ulcers, falling ill frequently, loss of appetite, distress, upset, brooding, blaming, guilt, loneliness, worries about family, occupational adjustment, economic crisis which are inclusive signs and symptoms of four important domains of QOL, which are physical functions, social functions, emotional functions, and financial issues.

#### Physical functions

Most of the doctors stated physical functions would not be affected much in cases of oral cancers unless they were stage 3 or 4 cases with radiotherapy.

“Postoperatively, pain probably would not be much of a problem; it gets relieved, post- surgery or post-treatment.” —Doctor 3

“I think the most commonly affected domain is the physical function.” “Right as I said, they should perform all the physical functions an oral cavity needs to perform.” —Doctor 7

We also asked about their view on why these are the most affected domains. Their views were:

“Yes, physical functions are mostly affected because anatomically, there are discrepancies in the tissues and structures. Second, there can be a physiological disturbance in their proper functioning. For example, pure surgery will not cause xerostomia or dryness of the mouth. But when coupled with radiation, particularly in stage 3 and 4 cases, recurrent cases, and xerostomia, it can be a very strong factor that causes physical problems with function. So, swallowing is primarily affected, which is one thing. Second, I feel that the food intake, they will not be able to take spicy food, which, probably in the Indian context, is very important.” —Doctor 8

“Yes, there are different domains related to QOL, like a few, say physical functions, patient experiences pain symptoms mainly, after postoperatively; if the stress is comorbid with this, then the patient can experience more intensity of the pain, because of the stress. So that thing can again hamper patients’ QOL.” —Doctor 9

“And functional loss, they cannot eat properly, they cannot swallow properly, they are dependent on all small things, probably, so that makes them suffer more.” —Doctor 10

#### Social functions

Oral cancer treatment often restricts patients’ social lives. Social determinants of health universally influence oral health outcomes and risk exposures. Disadvantaged groups face higher disease risks, especially in developing countries with poor living conditions. Tooth loss, oral cancer, and severe periodontal disease disproportionately affect deprived populations. Even in wealthier nations, underprivileged people experience high oral disease burdens and unmet needs. Lower social classes show elevated oral cancer incidence and mortality rates. These disparities stem from differential risk exposures tied to social class, including education levels, working conditions, sexual behaviors, biological agents, and habits like poor diet, tobacco use, alcohol consumption, and sedentary lifestyles. Barriers to early detection and control persist. Furthermore, patients’ social involvement diminishes due to aesthetic changes, oral cancer stigma, and guilt. In our study, physicians reported these challenges as described by their patients.

“Social functions are primarily related to his quality of speech and his facial appearance. There should not be scars or limited mouth openings which hinder them from proper talking or taking food in groups or in masses together.” —Doctor 10

“Social functions, because of postoperative cosmetic changes, patients try to avoid social programmes, avoid meeting their friends, and try to avoid their faces with a handkerchief. They do not want to tell anybody about their cosmetic issues & they have undergone cancer surgery. So, overall, their social functions get affected after treatment.” —Doctor 11

#### Emotional functions

Emotional burden or psychological issues are very common among oral cancer patients. Cancer itself is a horrifying disease for the patients. Dealing with cancer, treatment, and postoperative life changes brings a burden on the patient and also on their family members. Few doctors undoubtedly agree that emotional functions are the most affected domains amongst QOL in oral cancer survivors:

“Emotional function, you see, it is the fear factor of recurrence. Probably it is true also, as when patients come to us, they are in stage 3 or stage 4 many a time, almost 80% of patients who come to us are probably stage 3 or stage 4, and the recurrence rate is quite high in stage 3 and stage 4. From this point, it appears that patients who take treatment die after 6 months or 1 year. So, this is socially known, everybody knows, some of the relatives have had the cancer, and they died in 3 months, they know, and these are emotionally broken patients, and fear of recurrence or losing their lives is always there.” —Doctor 3

“Next is emotional functions, definitely, a patient’s cancer is an end-stage disease, where the patient thinks that their life is going to end soon. So, they undergo a tremendous range of emotions, like anxiety, anger- why this is with me, there is guilt that I have consumed tobacco and alcohol for so many years, and now I am suffering from this. Different patients’ emotions are different, like which stage they are, guilt, anger.” —Doctor 12

According to a few doctors, who were confident in their surgical treatment modalities, reconstruction of the lost part of the jaw, i.e., maxilla or mandible, helps patients regain their confidence. However, this is possible in early diagnosis of oral cancer, where the distant metastasis has not occurred, and reconstruction of the face gives patients good functional results, which indeed lead to a positive emotional state.

“I think the most commonly affected domains are the physical function and social function. I mean that can come in that sequence, first is physical function, second social function as related with the cosmetic appearance of his face and head neck area, third is emotional function where he has got some fear at the back of the mind that there will be recurrence at the local site or spread to the distant sites.” —Doctor 4

#### Financial issues

One of the major issues that oral cancer patients deal with is the financial aspects. Surgeries, reconstruction, chemotherapy, radiotherapy, and prostheses are all expensive in the context of the Indian scenario. Looking at the prevalence of oral cancer, mostly affected patients belonged to poor economic conditions that are prone to substance abuse habits, and later, the disease creates a treatment burden. Doctors have shared their experiences as:

“Coming to the Indian scenario, where we have very poor patients, probably healthcare management is expensive for them, and finances are limited. These patients come from a poor socioeconomic background; a major chunk of patients are from underprivileged areas, slum areas; they are totally broken up. They cannot make ends meet with these things, and so they delay their treatment. They wait for the social charitable hospital, where the queue or waiting is a very long period.” —Doctor 9

“Yes, if you see in the Indian context, oral cancer is mainly due to chewing of tobacco, and this tobacco chewing is mainly seen in lower socioeconomic status. So, in lower socioeconomic status, there is already financial lack, and cancer treatment is relatively costly for them, so a financial crisis is already there. Because of that, patients delay the treatment and overall QOL of the person, and the family also gets affected.” —Doctor 6

“Yes, definitely. I think in the Indian context, or oral cancer in Indian patients, the majority of patients who get cancer have poor socioeconomic status. Because the habits are more prevalent among people of low socioeconomic status. So, it definitely makes an impact on choosing a quality treatment or doctor.” —Doctor 2

### Theme 3: patients’ needs

QOL, satisfaction, and needs are distinct yet interrelated concepts that warrant separate measurement. Correlating health needs with health-related QOL scores offers benefits for routine clinical assessments targeting comprehensive care. In cancer patients, relationships among health needs, care satisfaction, and QOL remain complex. This research urges a reassessment of needs assessment and a deeper exploration of its ties to outcomes such as clinical endpoints, QOL, and care satisfaction. Under the ‘support’ category, physicians identified patient needs as financial help, health education, and family support.

#### Support

Support encompasses integrated aspects of oral cancer patients’ needs. Financial requirements stand out due to poor socioeconomic conditions. Doctors note that patients access free or low-cost treatment at public hospitals, but postoperative challenges arise from the need to continue ongoing medications and maintain nutritious diets, which patients must fund. In most cases, when the male family breadwinner develops oral cancer, financial support becomes essential for postoperative care.

“Financial support through charity, NGOs, or Government schemes should be made available to these needy patients.” —Doctor 4

“In the Indian scenario, the per capita income of people approaching us is very low, which means you can say 70–80% people with low socioeconomic status approach us with cancer. Some patients who approach us are at late stage of cancer, so there are multiple settings of chemo, radio, whatever the patient requires. So, there is a significant financial loss because the government does not provide comprehensive treatment; patients need to seek private therapy. So, definitely there is a financial crunch with the family with the treatment.” —Doctor 8

Secondly, family support, which most of the patients get, as oral cancer is not a contagious disease. Doctors reported that the patient’s caretaker or relatives were mostly positive about the patient’s oral cancer care.

“They are very supportive. There is one area I will say if there is a patient of tuberculosis, or AIDS, or a viral infection, cancer has got an upper hand in that. The patients’ relatives, they do care for them.” —Doctor 1

“I think, definitely, they are supportive. Because he is not a normal individual, in the sense that he is not very much derailed, but may be psychologically or physically, particularly in food intake and all, he may not be an absolutely normal individual. Not all the patients, but if their family members support, the patients do very well, particularly their diet, which should not be spicy, a diet that is exactly oriented for them, but in liquid form. That makes a difference.” —Doctor 7

“It depends upon the interpersonal relationships of the patient with family members. If interpersonal relationships are good, then family members are supportive. If the patient’s interpersonal relationships with family members are poor, they are fed up with the illness, and no support from family members. Because family members are also in the denial phase of not accepting the illness of the patient, so that is the issue that comes with relatives or caretakers.” —Doctors 11

#### Health education

Health education was considered an important aspect in postoperative care. Starting with avoiding or completely banning the substance abuse in day-to-day life care, good diet and nutrition were all essential to patients in terms of health education.

“Once operated, once treated, they probably take meticulous care. Whatever we tell them, be it a specific diet, a high vitamin diet, or a high protein diet, they follow it. Half of the time, when they are treated, half of their teeth are gone, half of the jaw is gone, or part of the face is gone; they do take care of whatever we tell them.” —Doctor 5

### Theme 4: barriers to a good quality of life

Barriers to good QOL were a crucial element that emerged from the interviews concerning doctors’ perspectives of oral cancer patients. Overall, the doctors acknowledged that patients should be guided in their autonomous decision to accept treatment. This guidance requires that doctors explain and justify a particular course of action before, during, and after the treatment. Although doctors have documentation about their patients’ clinical histories, to facilitate autonomous decision-making, it is important to make patients aware of treatments that, in their view, worked or did not, as well as whether they have heard anything positive or negative about treatments they have not yet tried. In particular, regarding clinical aspects and the insecurities that occupy patients’ minds, doctors have the following opinions.

#### Clinical aspects

Moreover, by identifying patients’ expectations regarding treatments, doctors can tailor their proposals to individual patients or explain to patients why their wishes are inappropriate. Paying attention to patients’ ideas and expectations about treatment is crucial to avoid nourishing false hopes, and to detect gaps between hopes and “what doctors can realistically offer.” Patients may be having trouble due to changes in their taste perception and should adjust their food habits.

“Not able to eat spicy food. Those who survive are unhappy because they have to eat bland food for a long time. We also advise them not to go for more spicy food. That is one of the most usual complaints.” —Doctor 12

They may have trouble with the prosthesis given. So, compliance with the treatment given was one of the barriers to good QOL.

“Yes, there is definitely. Because the prosthesis itself is difficult to maintain in a radiated, rehabilitated jaw, it probably hurts, and we do give guide planes to settle the occlusion, but compliance is always less. Patients will not be happy with the prosthesis.” —Doctor 9

While other doctors opined that “If you implement the reconstructive surgery or microvascular reconstruction of the area, definitely the prosthesis part is eliminated, and problems associated with the prosthesis are eliminated. So, they have to go to normal things.” “Prosthesis may not be that expensive. But the comfort that you get after reconstruction of the particular area is much different from having an artificial element. That’s how I look at it.” —Doctor 4

Patients are required to accept the treatment, according to the doctor’s perspective. Due to financial constraints, patients avoid attending recall visits with their doctors. However, doctors are supposed to give reminders about follow-up.

“Yes, that is definitely true. That is 100%.” “In my practice, all our patients do come for follow-up as and when we call them. First, for the first 6 months, monthly visits and after that, 3 months later, they do come for a check-up.” —Doctor 1

#### Insecurities

Fear of recurrence was a point mentioned by the doctors that could be a barrier to good QOL among patients. Doctors noticed these beliefs during follow-up visits that patients were under this fear.

“And side by side, these financial issues create emotional issues along with other issues like acceptance of illness, dealing with the emotions of accepting, denial is another issue, which is turning into different disorders like depressive disorder, anxiety disorder, somatoform disorder, you can say patients are presented with these symptoms.” —Doctor 12

“Patients were worried about getting back to their jobs, their physical capacity to work again. Some relatives might tell them, or they come to know about the recurrence of another patient.” —Doctor 4

Doctors said that they themselves usually do the counseling to motivate the patient to combat their postoperative battles as oral cancer survivors. A few of them refer to the psychiatrist for further counseling if they see the patient has signs and symptoms of anxiety, fear of death, or depression.

“Very frequently, we send our patients for psychological counseling. At least for the initial postoperative phase of 2 or 3 months, they do need psychological counseling and even sometimes an anti-psychotic drug, we have to put them on, because they are always depressed.” —Doctor 10

“Yes. Few patients are sent to the psychiatrist, few we counsel them, we ask them, their relatives, some counselor or some healer is definitely required.” —Doctor 2

“I do a counseling part. For example, making them aware that this is probably an irreversible change, e.g., related to the xerostomia, either it takes a very long time, or you adapt to that in your lifestyle.” —Doctor 7

### Theme5: overcoming barriers

Doctors suggested ways to enhance postoperative QOL for oral cancer survivors. Three key categories emerged: advocacy for health education, coping mechanisms, and regular check-ups. Sub-themes included advocacy, joint guidelines, joint planning, health promotion, health education, and dietary guidance. These address doctor-patient relationships, gaps in information on drug effects, protocols, alternative treatments, complementary medicine, and post-therapy follow-up. Such measures foster positive attitudes, beliefs, and experiences from cancer survivorship. For some, treatment end brings unexpected anger, sadness, or depression. Others feel fear mixed with hope, purpose, or opportunities for transformation, growth, and empowerment—often alongside vulnerability. Treating physicians must address these empathetically to support emotional recovery.

#### Advocacy for health education

Doctors responded positively when asked about the need for health education for postoperative care for oral cancer survivors and improving QOL. Health education, according to them, starts with the diagnosis of oral cancer stage, and extends to postoperative care. They also insisted upon this oral cancer survivor’s moral duty to discourage other people in their surroundings from continuing substance abuse, while they themselves abstain from it in due course. Patient should be educated about extra care to maintain oral hygiene as their oral health becomes vulnerable after surgery and radiotherapy.

“Yeah, definitely, they should be made aware right from the beginning that these particular habits, how detrimental they are, how they can convert into oral cancer, and another most important factor is that after surgery or after radiotherapy, they should be taught how visits to the dental surgeon or oral surgeon are important. Some patients whose jaws have been preserved or reconstructed, their dental rehabilitation is achieved by putting implants or a prosthesis, so they should be made aware to get the best QOL.” —Doctor 10

“There is a need for health education on all strata of the community. From primary to tertiary prevention, there is a need at every level. We should educate people regarding preventive aspects of cancer, like tobacco chewing and related stuff you get in the market, smoking, alcohol, and those things which are predisposing factors for cancer.” —Doctor 5

“General health education, you see at least every day in school-going children, one thing to tell them, twice brushing should hammer home to them. My smaller kid is in the second standard. If I tell him to brush at night, he probably will not do it, but if his teacher or a book says that he should brush twice, and they agree. In the same way, avoidance of habits like tobacco, related supari, betel nut, and gutkha should be avoided. It should be banned.” —Doctor 6

#### Coping mechanism

Despite scientific advances, uncertainty remains an inherent and significant issue in clinical practice. Among patients with advanced cancer, uncertainty about the future can lead to distress, loss of sense of control, and lower QOL. Helping patients manage uncertainty is a core domain of patient-centered care. Palliative care providers use validating strategies to help patients acknowledge and cope with the inherent uncertainty about their future. These methods can be used to develop communication training and guidelines for use with advanced cancer patients. Psychological counseling or treatments are essential for oral cancer patients to cope with the condition.

“When a patient comes to us for psychological counseling after an evaluation done by a cancer surgeon, we first see if the patient has a psychological disorder such as comorbid depression, psychosis, somatoform disorder, or anxiety. We plan the treatment, including pharmacological and non-pharmacological treatments. In pharmacological treatment, we start with the diagnosis and select the appropriate drug accordingly. In non-pharmacological treatment, counseling has a role. In counseling, the first step is supportive counseling, followed by belief-based counseling for the person. In short, I would like to tell the patients that we teach them how to accept the illness and the treatment. We usually teach patients how they should cope with their daily hassles, to live a good & healthy life.” —Doctor 8

Current clinical guidance suggests that all healthcare professionals should be able to provide basic psychological support, but promotes a tiered approach in which increasing professional expertise in psychological care should be sought as problems become more severe or complex.

“Because of the lack of mental health professionals in India, that sub-specialty called… onco-psychology…. is not highlighted yet…. So definitely, there is a need for special counselors in this area to work with the cancer patients, like oral cancers, also like where there are centers where a lot of cancer patients are coming or getting operated, there should be a counselor setting, and their patients should be followed strictly by them along with their family also.” —Doctor 2

Diet and nutritional counseling form an important part of postoperative care instruction. The re-functioning of lost body tissue, healing, surviving chemotherapy, radiotherapy, coping with changes in taste, and boosting immunity are possible with a good diet and nutrition. Many doctors said that they advised their patients to consult a dietitian or nutritionist available at their hospital for specific advice.

“Once operated, once treated, they probably take meticulous care. Whatever we tell them, whether it’s a specific diet, a high-vitamin diet, or a high-protein diet, they follow it. Half of the time, when they are treated, half of their teeth are gone, half of the jaw is gone, or part of the face is gone; they do take care of whatever we tell them.” —Doctor 3

“Of course, they should resume the diet that has more nutritional values, particularly a good protein diet and a diet that has antioxidants built in and multivitamins. Milk, green leafy vegetables, less of the carbohydrates and fats, and more of protein, and a vegetarian diet.” —Doctor 11

A few doctors also stated that a good surgical procedure is a key factor in helping patients cope with the appropriate surgery.

“I think good surgical procedure is the key factor in having a good QOL that may be physical, social, emotional, and everything. If a very good quality surgery is done, which includes proper resection addressing the neck for cervical metastasis; most importantly, reconstructive surgery, it would definitely help these patients to give them an excellent QOL.” —Doctor 9

Societal acceptance is an important issue that patients should be encouraged to face. Patient should be advised to be confident in their current situation and optimistically face society. They should resume their work as early as possible. They should attend social programs earlier. These things would help to maintain good mental health with a positive attitude.

“First, there should be preoperative counseling of the patient, which is a must, regarding how the illness is, what the treatment is, duration, prognosis, and all should be explained to the patient in their local language so that they will understand and prepare themselves for future outcomes. Postoperatively, the patient should be counseled about cosmetic changes they will experience and any further social issues they may face. So, our role is to make the patient more comfortable, or he should be able to cope with the situation, whatever comes his way.” —Doctor 6

#### Regular check-up

It is clear, then, that oral cancer screening helps identify patients at earlier stages, a finding that can be attributed in part to the limitations of current treatment methods. The oral screening examination can reduce the risk of advanced cases and is also associated with reduced oral cancer mortality. This is true for the recurrence of oral cancer also. The patient must follow the regular recall visits as advised by the doctors. As patients reported that fear of recurrence was one of their insecurities, regular visits to the doctors or dentists can only help them alleviate this fear.

“Recall visits are important… First one or two years, they are very regular, every month, then we schedule them every 3 months, so the follow-up protocol is very good. We will be able to detect early if there are recurrences.” —Doctor 4

“The most important thing is their regular check-up & follow-ups. This is important because these diseases, despite good surgical work or proper treatment, have a chance of recurrence. And second is related to their habits. Naturally, they should now discontinue all their habits which have been blamed for causing oral cancer, particularly tobacco and in the Indian context, the pan masala and gutkha habits.” —Doctor 12

“Fear of recurrence, we tell that a good recall or follow-up will detect the early change. For patients who can afford it, we ask them to perform PET Scans, maybe after 6 months to one year. As of today, all our patients are comfortable and do not require a PET scan.” —Doctor 7

“Depending upon the diagnosis and the need for counseling, the sessions are planned. If there is a definitive diagnostic disorder along with the cancer, I mean, during cancer or postoperatively, we call for frequent visits. However, the follow-up rate is poor in these patients. Because already they are undergoing financial issues and paying for counseling sessions is again a burden, and so they feel there is no need.” —Doctor 1

“Early treatment or check-up. One thing we can tell our patients is to come to us early. We would be able to give these patients a better life and better function, improving their QOL; we can exactly replicate and reconstruct and give them, like after amputation, the Jaipur foot and all, so that people can work and earn. In the same way, we can rehabilitate our patients with free flaps, with early stages we can replace a bone with a bone, and we can replace the tooth with an implant, which will give them an almost normal life, not 100%, but at least 90–95% functions they can achieve.” —Doctor 5

Table 1 summarizes the themes and subthemes with representative quotes.

**Table 1:** Summary of themes, subthemes, and representative quotes.

Themes	Sub-themes	Quotes
Understanding of the quality of life	Concept Definition	“I think what I mean or what I understand by quality of life of patients who have been operated for cancer is related to their comfort or physical comfort related to their day-to-day functions.”
Affected domains of quality of life	Physical functions Social functions Emotional functions Financial issues	“I think the most commonly affected domain is the physical function, and second, the social function. I mean that can come in that sequence, first is physical function, second social function as related with the cosmetic appearance of his face & head neck area, third is emotional function where he has got some fear at the back of the mind that there will be recurrence at the local site or spread to the distant sites.”
Patients’ needs	Support Health education	“If their family members support, the patients do very well, particularly their diet, which should not be spicy, a diet that is exactly oriented for him, but in liquid form. That makes a difference.”
Barriers to a good quality of life	Clinical aspects Insecurities	“Patients were worried about getting back to their jobs, their physical capacity to work again. Some relatives might tell them, or they come to know about the recurrence of another patient.”
Overcoming barriers	Advocacy for health education Coping mechanism Regular check-up	“Early treatment or check-up. One thing we can tell our patients is to come to us early. We would be able to give a better life and better function to these patients to improve their quality of life, and we can exactly replicate and reconstruct and give them, like after amputation, the Jaipur foot and all, so that people can work and earn.”

## Discussion

This qualitative study explored treating physicians’ perceptions of postoperative QOL among oral cancer survivors in Aurangabad, Maharashtra, a region with a high burden of smokeless-tobacco–related disease. Consistent with international research, physicians conceptualized QOL as a multidimensional construct, shaped by interrelated domains of physical functioning, psychosocial wellbeing, emotional stability, and socioeconomic security. Their descriptions mirrored established frameworks that emphasize survivorship as a complex adaptive process encompassing functional recovery, social reintegration, and psychological adjustment (19, 20). The emergence of themes such as “Understanding of QOL” and “Affected domains of QOL” reflects the complex, multidimensional nature of oral cancer survivorship within India’s resource-constrained healthcare context, where fragmented care pathways often prioritize tumor control over holistic wellbeing, leading physicians to recognize functional impairments (e.g., speech, swallowing) as dominant concerns while sometimes overlooking psychosocial needs.

### Physical and functional recovery as foundational to QOL

Physicians emphasized functional impairments, including trismus, dysphagia, xerostomia, and mastication difficulty as primary determinants of diminished QOL. These findings align with postoperative studies demonstrating sustained deficits in swallowing, chewing, and oral function after ablative surgery or chemoradiation (7, 8, 21). Several clinicians highlighted the role of reconstructive strategies and timely prosthetic rehabilitation in improving postoperative function, echoing evidence that microvascular free-flap reconstruction, dental implants, and CAD/CAM prostheses markedly enhance oral-health–related QOL (11, 22, 23).

### Emotional and psychological distress as persistent sequelae

A dominant insight from clinicians was the high prevalence of emotional distress—particularly fear of cancer recurrence (FCR), anxiety, depression, and body-image concerns. These perceptions are consistent with reports identifying FCR as among the most distressing and persistent symptoms in head and neck cancer survivors (24, 25). Clinicians recognized the need for structured psychological support, yet noted the limited availability of psycho-oncology services in the region. This mirrors challenges observed in similar low-resource contexts, where psychological morbidity remains under-detected and under-treated (26, 27).

### Socioeconomic constraints as structural determinants of QOL

Physicians repeatedly described financial toxicity as a major barrier. Many survivors were primary earners from lower-income households, facing substantial out-of-pocket expenditures for surgery, radiotherapy, medications, prostheses, imaging, and travel. These insights mirror Indian research demonstrating that financial strain predicts poorer adherence, delayed follow-up, and lower QOL (28, 29). Such findings reinforce the need for health-system reforms, including subsidized prosthetic rehabilitation, expanded insurance coverage, and institutional financial navigation services.

### Gaps in survivorship pathways and multidisciplinary care

Clinicians acknowledged the absence of structured, multidisciplinary survivorship pathways integrating dental and prosthetic rehabilitation, speech and swallowing therapy, psychological services, and nutritional counseling.

This gap contrasts with international survivorship models emphasizing coordinated, long-term follow-up and rehabilitation across disciplines (19, 30). Physicians also noted challenges with prosthesis use in irradiated tissues, reflecting broader evidence that patient education, iterative adjustments, and post-prosthetic follow-up are critical for successful rehabilitation (31).

### Communication, counseling, and the doctor–patient relationship

A recurring observation was the importance of clear, empathetic communication. Physicians noted that preoperative counseling and expectation-setting improved postoperative coping. This is consistent with research highlighting argumentation-based communication as a tool to enhance shared decision-making and reduce misalignment of expectations (32). Many clinicians provided informal counseling but noted that systemic provision of psychological and educational support remains insufficient. Divergences in physician perspectives were evident, with surgical specialists emphasizing physical rehabilitation barriers and psychiatrists highlighting emotional functioning gaps, underscoring the need for specialty-specific training in survivorship care.

### Implications for practice and policy

Findings suggest several actionable priorities: Incorporating validated QOL instruments in postoperative clinics can systematically identify deficits requiring intervention. Hospitals should develop structured pathways that integrate prosthodontics, nutrition, psychology, and rehabilitation services tailored to local capacity. Policy-level reforms—including insurance expansion, public–private partnerships, and subsidized prosthetic programs—are essential to mitigate financial toxicity (33). Education on tobacco cessation, oral hygiene, nutrition, and adherence to follow-up should be strengthened through culturally informed interventions. Training clinicians, dentists, and allied health staff in survivorship care, rehabilitation protocols, and psychological first-line support is crucial for implementation. Identified gaps, including inadequate multidisciplinary coordination and limited psychosocial support, could be addressed through practice changes such as implementing standardized survivorship care plans, routine QOL screening in follow-up clinics, and integrating mental health services into oncology protocols to foster patient-centered care.

### Strengths and limitations

A key strength of this study is its focus on clinician perspectives from a high-burden district in India, offering contextual insights that complement patient-based QOL research. While psychiatrists may emphasize psychological dimensions more than surgical specialists such as oral and maxillofacial surgeons or oncologists, the multidisciplinary composition of our sample—encompassing diverse specialties—strengthens the generalizability of findings by reflecting real-world clinical perspectives in comprehensive oral cancer survivorship care. Future studies could employ stratified analyses by specialty to further elucidate these disciplinary differences. Rigorous framework analysis and data triangulation enhance credibility. Limitations include geographic concentration in Aurangabad, potential social desirability bias, and lack of matched patient-reported data. All participating physicians in this study were from private practice settings in India, which may influence their perceptions of QOL domains due to differences in patient demographics, resource availability, and healthcare access compared with public-sector facilities. A key limitation of this study is the relatively small number of participants in each specialty, which may limit the depth of insights specific to individual professional perspectives. These limitations highlight opportunities for future research to enhance transferability across geographical contexts, validate findings with patient-reported data, explore public sector perspectives, and deepen specialty-specific insights, thereby building on the foundational themes identified in this study. Future mixed-methods research integrating clinician and survivor perspectives may yield a more comprehensive survivorship model.

## Conclusion

This study showed that treating physicians in Aurangabad view postoperative QOL for oral cancer survivors as inherently multidimensional, shaped by interlinked physical, emotional, social, and socioeconomic influences. Functional difficulties with swallowing, chewing, speech, and dry mouth were seen as core problems that frequently precipitate wider psychological distress and social withdrawal. Physicians described high levels of fear of recurrence, anxiety, depression, and body-image concerns, highlighting substantial unmet needs for ongoing, accessible psychological care. Socioeconomic hardship and financial toxicity were recognized as key constraints on access to optimal reconstruction, prosthetic rehabilitation, nutrition, and consistent follow-up, compounded by fragmented survivorship pathways and limited multidisciplinary coordination. These findings point to an urgent need for structured, context-appropriate survivorship models that incorporate routine multidomain QOL assessment, timely prosthetic and nutritional support, strengthened psychosocial services, targeted patient–family education, and policy measures to reduce financial burden. Building capacity within clinical teams for rehabilitation and mental health support is crucial. Overall, coordinated, context-sensitive strategies spanning clinical practice, community engagement, and health-system policy are essential for improving long-term function, emotional wellbeing, and social participation for oral cancer survivors in low-resource settings.

## Data Availability

The raw data supporting the conclusions of this article will be made available by the authors, without undue reservation.
